# Overcoming the Challenges Imposed by Humoral Immunity to AAV Vectors to Achieve Safe and Efficient Gene Transfer in Seropositive Patients

**DOI:** 10.3389/fimmu.2022.857276

**Published:** 2022-04-07

**Authors:** David-Alexandre Gross, Novella Tedesco, Christian Leborgne, Giuseppe Ronzitti

**Affiliations:** ^1^ Genethon, Evry, France; ^2^ Université Paris-Saclay, Univ Evry, Inserm, Genethon, Integrare research unit UMR_S951, Evry, France

**Keywords:** AAV vectors, gene therapy, immunogenicity, humoral response, B-cells, neutralizing antibodies

## Abstract

One of the major goals of *in vivo* gene transfer is to achieve long-term expression of therapeutic transgenes in terminally differentiated cells. The extensive clinical experience and the recent approval of Luxturna^®^ (Spark Therapeutics, now Roche) and Zolgensma^®^ (AveXis, now Novartis) place vectors derived from adeno-associated viruses (AAV) among the best options for gene transfer in multiple tissues. Despite these successes, limitations remain to the application of this therapeutic modality in a wider population. AAV was originally identified as a promising virus to derive gene therapy vectors because, despite infecting humans, it was not associated with any evident disease. Thee large proportion of AAV infections in the human population is now revealing as a limitation because after exposure to wild-type AAV, anti-AAV antibodies develops and may neutralize the vectors derived from the virus. Injection of AAV in humans is generally well-tolerated although the immune system can activate after the recognition of AAV vectors capsid and genome. The formation of high-titer neutralizing antibodies to AAV after the first injection precludes vector re-administration. Thus, both pre-existing and post-treatment humoral responses to AAV vectors greatly limit a wider application of this gene transfer modality. Different methods were suggested to overcome this limitation. The extensive preclinical data available and the large clinical experience in the control of AAV vectors immunogenicity are key to clinical translation and to demonstrate the safety and efficacy of these methods and ultimately bring a curative treatment to patients.

## Introduction

Adeno-associated viruses (AAV) are constituted by a 25-nanometer protein icosahedral capsid containing a single-stranded DNA genome flanked by two palindromic inverted terminal repeats (ITR). The 4.7 Kb AAV genome encodes for four different Rep proteins (Rep78, Rep68, Rep52 and Rep40), three Cap proteins (VP1, VP2 and VP3), the assembly activating protein (AAP) and the newly identified membrane-associated accessory protein (MAAP) (1–3). Cap proteins constitute the capsid of the virus and mediate the interaction with the host. The capsid proteins VP1 and VP2 share most of the sequence with VP3 that is the major component of the AAV capsid with 50 out of 60 capsid subunits being VP3 (4). At the time of writing, 13 different AAV serotypes and more than hundred isolates, distinguished by amino acid modifications in the capsid proteins have been identified in different species (1, 5–8).

After its isolation as a contaminant of adenovirus preparations in 1965 (9–11), it took almost 20 years for molecular cloning of the AAV genome thus opening the way to the generation of recombinant AAV (rAAV) vectors from AAV by encapsidating a transgene expression cassette flanked by the ITRs from serotype 2 (12–15). Importantly, through this process, the same transgene expression cassette can be pseudo-typed by virtually any of the natural AAV serotypes. As for the natural virus, capsid composition affects the tissue tropism and the intracellular trafficking of the recombinant virus (1, 16).

The adenovirus-free method of rAAV production is based on transient transfection of mammalian cells with three plasmids (17). Two of the plasmids provide in trans the *rep* and *cap* genes and the helper genes, typically from adenovirus (18, 19). A third plasmid contains the transgene expression cassette flanked by the two ITRs. Recombinant AAV vectors can be produced in mammalian cells also through the infection with adenovirus (20) or herpes simplex virus (21). Finally, rAAV can be produced in insect cells infected with baculoviruses carrying all the components necessary for vector production (22). Regardless of the production method, and differently from the wild-type virus (23), rAAV vectors are produced as a mix of full capsids, containing the genomic material, and empty capsids. Several distinct natural serotypes isolated in humans and other mammalian species were produced as well as chimeric AAV obtained through different techniques [recently reviewed in (24)].

As previously mentioned, the transduction properties of rAAV vectors are a direct consequence of the capsid composition. Surface-receptors binding, endocytosis and intracellular trafficking as well as the escape of the vector from the late endosome/lysosomal compartments and the nuclear import contributes to the preference of rAAV vectors for a certain cell type/tissue [reviewed in (25)]. After nuclear translocation, the genome of rAAV do not integrate efficiently and remains in the episomal form (26).

AAVs infect humans and other mammalian species starting from the first years of life (27–33), but are not associated to any known disease (34). Infection with AAVs results in the formation of a humoral response against the virus. Although the frequency of individuals seropositive to AAV may vary, large portions of the human population are infected, with an estimated seroprevalence for neutralizing antibodies (NAb) for the different AAV serotypes in the range of 30-60% (27–29, 35–37). The presence of NAbs due to exposure to the wild-type AAV reduce the number of patients that may benefit from the treatment.

Although multi-year transgene expression was reported in large animals and patients treated with rAAV (38–43), loss of expression at long-term is still possible as a consequence of slow cell replication or mechanisms of inactivation acting on the vector genome (44). The formation of anti-AAV NAbs with long persistence and wide specificity after injection of rAAV (45) represents an important limitation to re-administration of the vectors in patients that have received the rAAV and experienced an expression loss.

**Figure 1:**
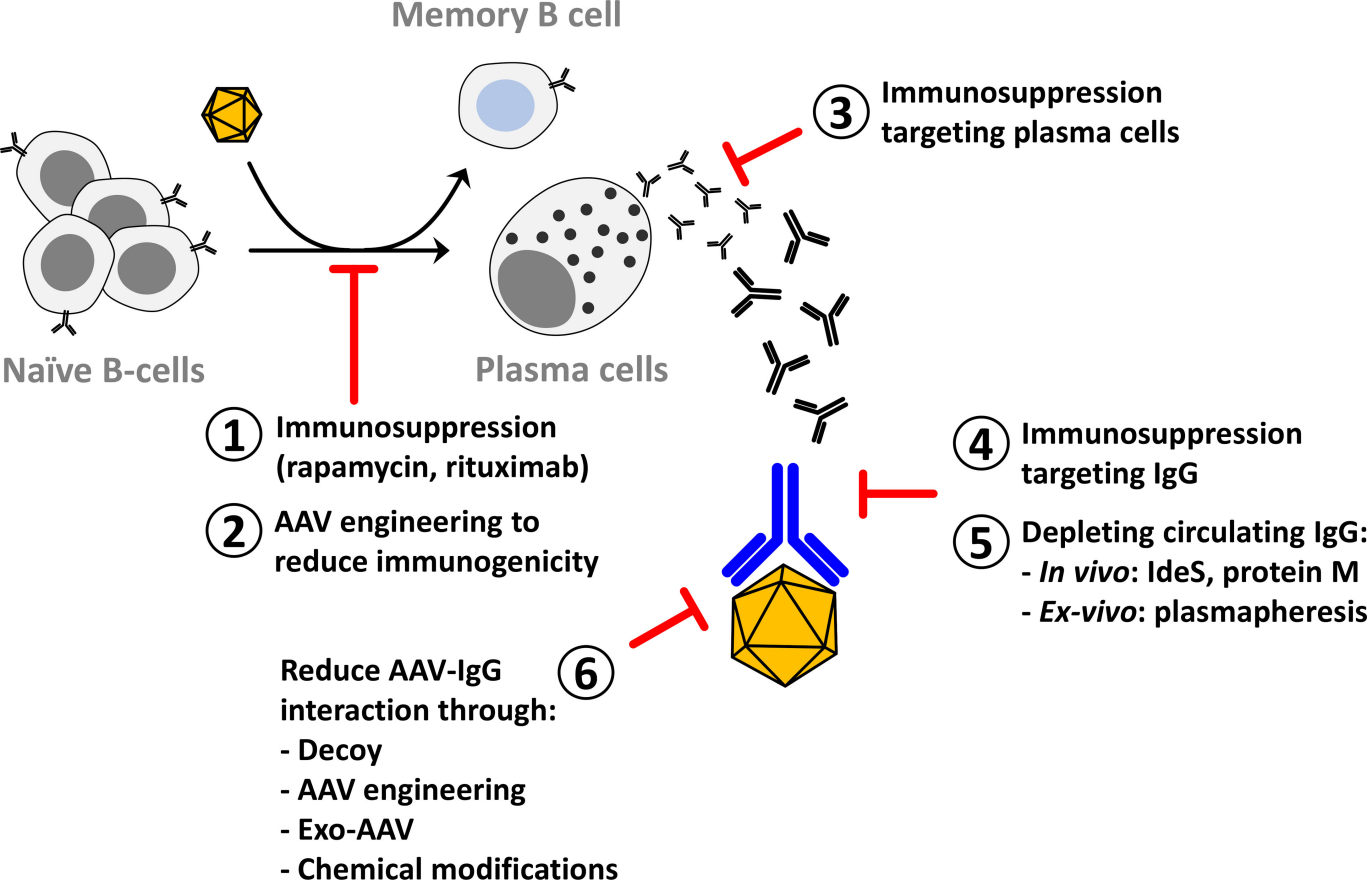
Methods to reduce the impact of anti-AAV neutralizing antibodies on AAV gene transfer. After AAV gene transfer in seronegative individuals naïve B-cells are activated to plasma cells that are specialized in antibody secretion. Part of the activated B-cells become memory B-cells that participate in the long-term stability of the humoral response. Methods to reduce the activation of B-cells include immunosuppression to target the CD4-mediated helper function (1) or to reduce the viability of plasma cells (3) and AAV engineering to reduce the activation of the immune system (2). Immunosuppression to reduce circulating IgG has been proposed in autoimmune diseases and can be applied in AAV gene therapy (4). Circulating IgG can also be reduced through *in vivo* or *in vitro* depletion (5). Another method to reduce the impact of anti-AAV IgG on gene transfer efficacy is to modify the vector to evade neutralization (6).

This review will then focus on the methods that overcome the limitations imposed by the humoral response to gene transfer with rAAV (**Figure 1**) perceived as fundamental to expand patients access to therapy and allow for life-long treatment of genetic diseases.

## Humoral Immunity to AAV

AAVs naturally infect humans and other mammalians starting from infancy. Although the natural history of AAV infection is still poorly characterized, data indicated that, after infection, a large proportion (ranging from 30% to 60%) of the infected population developed cross-reactive anti-AAV NAbs possibly due to successive infections and/or broad cross-reactivity between AAV serotypes (46). The proportion of seropositive individuals increased from early childhood to peak at the age of adolescence (27–29). Infection with wild-type AAV resulted in antibodies from all IgG subclasses with a preference for IgG1. The levels of IgG, in general, correlated with the neutralizing antibody titers measured by an *in vitro* neutralization test (35, 36) although it was reported the presence of IgG that while binding to AAV vectors did not neutralize their capacity to enter the cells *in vitro* and *in vivo* (47). NAbs against AAV2 and AAV1 were reported as the most prevalent in the human population (48) starting from three years of age. Importantly, the occurrence of maternal transfer of Nabs, with neonates being seronegative only between 7 and 11 months, leaves a short time window for AAV gene transfer (27).

At the very beginning of gene therapy with AAV vectors, the role of pre-existing immunity in gene transfer efficacy was uncertain. The presence of neutralizing antibodies to AAV2 was not among the exclusion criteria in the first clinical trial of liver gene transfer after systemic infusion with this vector (49). Although the choice was possibly motivated by the use of a ‘‘local’’ administration of the vector, i.e. portal vein infusion, data showed a robust reduction in transgene expression in one patient having low anti-AAV neutralizing titers (49). Preclinical data obtained in mice and published in the same year supported this finding (50). Later on, experiments in non-human primates (NHP) clearly showed neutralization due to low-titer anti-AAV pre-existing antibodies (51). Based on this experience, exclusion criteria for seropositive patients were included in most of the clinical trials.

In humans, administration of AAV vectors resulted in a fast and robust rise in IgM, followed by IgG with a high neutralizing titer either after intramuscular (52) or portal vein infusion (49). Similarly to the wild-type virus infection, the neutralizing titers in general correlate with the levels of binding antibodies as measured by ELISA (53). Long term follow-up of patients receiving treatment for hemophilia B indicates up to fifteen years of stability of neutralizing anti-AAV antibody titers for the injected AAV (43, 45). Importantly, while higher titers were measured against the injected serotype, the antibodies were neutralizing also against other serotypes although with lower titers (45).

Neutralizing factors, capable of binding to AAV vectors thus reducing their efficacy *in vitro* were identified as elements of the complement system (54, 55). Important and open questions about neutralizing factors against AAV are their exact nature and role in the immune response and gene transfer efficacy *in vivo*. Complement activation was reported in ongoing clinical trials for Duchenne muscular dystrophy and spinal muscular atrophy type 1, in which patients administered with high-dose AAV vectors developed serious adverse events including liver dysfunction, acute kidney injury or thrombocytopenia (56–58). More recently, LogicBio reported two cases of thrombotic microangiopathy following the administration of 5 x 10^13^ vg/kg of AAV-LK03 (59). Both complement proteins and antibodies are likely being investigated for their role in those adverse events. In animal models, neuronal degeneration in the dorsal root ganglia (DRG) were observed few weeks after high-dose intravenous or intrathecal administration in piglets and NHPs (60–62). This neuronal toxicity was not prevented by conventional steroid regimens and was dependent on overabundance of the transgene product and subsequent activation of cellular stress pathway (63). Beside this DRG toxicity, acute liver damage occurred in NHPs 3-4 days after high-dose systemic delivery of AAV9 or AAV-PHP.B, with acute elevations in liver enzymes, thrombocytopenia as well as acute hemorrhage (60, 64). Interestingly, prophylactic steroid treatment mitigates the increase in liver enzymes but not the thrombocytopenia suggesting that these two events may not correlate. Moreover, selection of seronegative NHPs for the studies, timing of this acute toxicity as well as activation of alternative complement pathway support a working hypothesis where classical complement pathway activated by antigen-antibody immune complexes is likely not involved in these deleterious events. Poor availability of predictive animal models to study complement activation and limited clinical experience with AAV vectors administration in seropositive patients may be two of the reasons why, so far, a mechanistic proof of the involvement of these factors in AAV toxicity is still missing.

Regardless of their involvement in unwanted toxicities, the long-term stability and the wide neutralization potential of the humoral immune response to AAV vectors are important limitations of this gene therapy modality as they preclude both vector infusion in seropositive patients and re-administration in patients that received a sub-optimal dose or in whom the efficacy of gene therapy waned overtime.

## AAV Capsid Modifications to Reduce Antibody-Mediated Neutralization

Early attempts to prevent capsid neutralization were based on the use of empty capsids as decoys to shield the full particles from neutralization (65). This approach required large amounts of empty AAV capsids thus increasing total vector load. Liver toxicity associated with the death of four patients was recently reported in a clinical trial for X-linked myotubular myopathy (NCT03199469) and likely linked to the high vector dose and pre-existing liver disease. Another potential limitation in the decoy strategy is the lack of efficient methods for the production and purification of empty capsids with limited encapsidation of random cellular components and genomic and plasmid DNA.

The relatively low complexity of the capsid structure and AAV virus genome has allowed for the creation of a multitude of engineered capsids with improved properties. Rational design as well as random mutagenesis and capsid shuffling were used to derive novel serotypes. Peptide insertion in specific VP3 protein position combined with directed evolution, provided a powerful tool to improve AAV capsids biodistribution (66–68). Structure-guided evolution was also used to modify the epitopes recognized by antibodies thus evading neutralization. In particular, the resolution of the structure of AAV2 complexed with a monoclonal antibody (69) enabled rational mutation of the AAV2 sequence to avoid neutralization from this specific clone (70). An evolution of this approach is based on the rational mutation of the regions of AAV capsids involved in the interactions with polyclonal human serums (71). Through this approach, novel AAV capsids were identified with improved evasion of NAbs without compromising vector productivity or biodistribution. Another method to isolate neutralization-resistant AAV vectors is the directed evolution of AAV libraries in an *in vitro* neutralization setting. Although limited by the poor *in vitro* transduction of AAV vectors, the use of selective pressure on randomly mutated or shuffled AAV capsid libraries led to the isolation of serotypes that were less neutralized by purified intravenous immunoglobulins (IVIg) (72–74). The use of immune-orthogonal orthologues of AAV has been proposed to overcome the constraints imposed by the humoral response to AAV vectors (75). This approach is limited by the higher complexity of the human immune system that reduces the chance to identify orthologs (76). However, after confirmation in relevant preclinical models, this approach may represent an option to dose seropositive patients.

Another interesting approach to evade antibody-mediated neutralization is the use of AAV vectors co-purified with exosomes derived from the producer cell line. Exosomes are extracellular vesicles naturally produced by various types of cells both in culture and *in vivo* and used by the cells for communication or exchange of proteins and genetic material (reviewed in (77)). Importantly, some viruses showed the ability to hack this system and acquire a membrane shield to the immune system. Similarly, AAV vectors associated with membranes of the producer cell lines can be purified as exosome-associated AAV vectors or exo-AAV (78). Exo-AAV showed improved cell transduction and reduced sensitivity to neutralization in an *in vivo* neutralization assay (79–81). One potential limitation associated to the clinical translation of exo-AAV is related to the potential adjuvant effect of the proteins and nucleic acid co-packaged with the exo-AAV and to the challenges regarding the development of a clinical grade manufacturing process and the associated analytical methods.

The high stability of the AAV capsid in extreme conditions led to the development of methods to modify the surface of AAV capsids by cross-linking different chemical molecules. RGD-containing peptides as well as PEG were originally proposed to improve AAV tropism but also to reduce the impact of neutralizing antibodies on transduction (82–86). More recently, amino sugars, known to increase liver targeting were chemically linked to the AAV capsid (87). Although the chemical alteration of AAV vectors may, in principle, extensively modify the capsid surface, only partial evasion of NAbs was reported so far (87). One important limitation of the chemical modification of the AAV capsid and more generally of proteins is that drastic conditions are required to obtain extensive modification of the surface, and this may not be compatible with the stability of the 3D structure of the virus.

Although different methods were proposed to modify the AAV capsid and reduce the impact of neutralizing antibodies, so far, there is no clinical proof of the possibility to administer those modified AAV vectors in seropositive individuals. Perhaps in this context are worth mentioning the recent clinical results suggesting that AAV5 is less sensitive to neutralization in humans (88). Administration of an AAV5 vector expressing human coagulation factor IX in patients who have retrospectively been shown to have significant NAb titer resulted in similar expression levels of the transgene with a lower impact on the transduction compared to what was previously reported for AAV2 (49). Those results were confirmed in a large study in NHPs were different doses of AAV5-hFIX demonstrated efficacy irrespective of the presence of pre-existing anti AAV5 NAb at titers up to 1:1030 (88). The absence of standardized tests to compare neutralizing titers measured in the different clinical trials limits any further conclusion. However, these results suggest inconsistency between the neutralization titers measured with an *in vitro* assay and the neutralization *in vivo* after administration of the AAV5 vector in humans. If confirmed in a larger number of patients, the use of AAV5 for liver gene transfer may represent a valid option to treat seropositive patients.

## Immunosuppression to Prevent Anti-AAV Capsid Antibody Formation

The formation of anti-AAV NAbs following AAV gene transfer is dependent on the serotype used and on the route of administration which may influence the presentation of the AAV capsid proteins on antigen-presenting cells (APCs) (89–91). Blocking of classical costimulatory pathways, implicated in B-cell activation, demonstrated efficacy on the inhibition of humoral response and allowed AAV re-administration in mice (92, 93). Early findings supported the involvement of the innate signaling in the formation of the humoral immune response to AAV vectors (94). In particular, loss of MyD88, a central node for the signaling downstream of Toll-like receptor (TLR) and interleukin-1 (IL-1) receptor pathways, significantly reduced anti-AAV NAb titers (95, 96). An isotype switch from IgG2c to IgG1 was also observed in MyD88 deficient mice possibly due to a switch to a Th2-type immune response in the absence of Th1-polarizing stimuli. In human peripheral blood mononuclear cells (PBMCs), stimulation with AAV2 capsid resulted in an interleukin-1b (IL-1b) -dependent B-cell maturation and anti-AAV antibody secretion (97). Importantly, the inhibition of IL-1b through monoclonal antibodies resulted in decreased anti-AAV antibody formation both *in vitro*, in human PBMCs and *in vivo* in C57Bl6 mice challenged with an AAV8 vector (97). Based on these data, the inhibition of innate signaling seems to be a promising approach to control adaptive immunity to AAV vectors. Another relevant target for the suppression of the anti-AAV humoral response is the inhibition of the CD4 co-receptor. Both the antioxidant MnTBAP and a non-depleting anti-CD4 antibody were used to reduce the activation of CD4+ T-cells, thus decreasing the anti-AAV humoral response (90, 98). As an alternative, rapamycin (Sirolimus), an immunosuppressive drug largely used in transplantation, was shown to reduce anti-AAV immune response (99). Interestingly, rapamycin formulated in nanoparticles showed greater efficacy in small and large preclinical models and allowed to re-administer an AAV vector in primates while reducing the dose of rapamycin by specific targeting APCs (100–102). B-cell depletion with an anti-CD20 antibody (Rituximab) has also shown efficacy in the reduction of anti-AAV humoral immune response in preclinical models (103, 104). The combination of Sirolimus and Rituximab is being investigated in a clinical protocol for AAV vector re-administration in Pompe disease (NCT02240407). The clinical protocol involves the repeated intramuscular administration of an AAV9 vector expressing GAA in the presence of an immunosuppression regimen combining Rituximab and Sirolimus. Although the results of this trial are not public, a single-case report indicated that simultaneous treatment with these two drugs prevented the formation of anti-AAV1 antibodies when the vector was administered into the diaphragm (105).

One important limitation of the immunosuppression methods described above is that proof of their efficacy was obtained mainly in naïve animals that received the AAV vector for the first time and their efficacy is likely very limited in primed animals were the B-cells are already activated and differentiated in both memory B-cells and plasma cells (PCs). Since PCs are responsible for the maintenance of high levels of circulating antibodies, different strategies, derived from those used in autoimmune diseases and myeloma, were attempted to substantially reduce the number of active PCs. Targeting PCs may result in toxicity and the debate on the risk/benefit ratio of such approaches in AAV gene therapy is still open. Among them, we may cite the use of bortezomib, a proteasome inhibitor approved for the treatment of mantle cell lymphoma and myeloma, that despite its toxicity, has been proposed for the control of anti-AAV immune response (106). Although the combination of bortezomib with AAV vectors was also tested in dogs (107), no significant effect was reported on the pre-existing humoral immunity to AAV vectors and the strategy was never moved used in the clinic. Novel approaches are being developed for the control of PCs proliferation with improved selectivity and reduced toxicity [reviewed in (108)] and based on their improved risk/benefit ratio they may be tested as a pretreatment to reduce the humoral immune response to AAV vectors and allow for vector administration in seropositive patients.

## Methods to Reduce the Levels of Circulating Antibodies

As already discussed, plasma cells are a relevant target to reduce the impact of neutralizing antibodies on gene transfer. However, the current approaches to drastically reduce the number of antibody-secreting cells suffer from toxicity and poor selectivity.

A strategy to reduce the impact of NAbs on AAV transduction relies on the isolated perfusion of the liver with a catheter to flush the blood from the liver (109, 110). Although this method allows to reduce the titers of NAbs and increase liver transduction in seropositive monkeys, its clinical translation may prove complex and potential inflammation of the liver related to the procedure may threaten the outcome of gene transfer in humans.

As an alternative, reduction of the circulating levels of IgG is an ideal strategy to reduce the impact of anti-AAV NAbs on AAV gene transfer. Among those methods, the use of blockers of neonatal Fc receptors (FcRn) was proposed for AAV vector administration in seropositive patients (111), although it was never tested in this setting. FcRn receptors have a profound effect on the levels of circulating IgG by increasing their recycling (112). Blocking the action of FcRn led to increased antibody trafficking to lysosomes and degradation and reduced the levels of circulating IgG in both NHPs and humans (113, 114). FcRn-targeting therapeutics demonstrated efficacy in preclinical models of autoimmunity such as antibody-induced arthritis, experimental autoimmune encephalomyelitis or immune thrombocytopenia [reviewed in (115)].

A more invasive alternative to FcRn blockers is the use of plasmapheresis. Plasmapheresis is widely used in autoimmune diseases to reduce the negative effects of auto-antibodies. Early findings indicated that multiple cycles of plasmapheresis were needed in humans to significantly reduce the level of anti-AAV NAbs in humans (116). In NHPs, two cycles of plasmapheresis allowed for muscle targeting after administration of AAVrh74 through isolated limb perfusion (117). More recently, a second study in NHPs demonstrated that three cycles of plasmapheresis reduced the levels of neutralizing antibodies to AAV5 of at least ten-fold on average and allowed for repeated administration of the same vector bearing two distinct transgenes (118). In the same publication, the authors showed that two cycles of plasmapheresis in humans led to a three to five-fold decrease in the anti AAV2 and AAV9 NAb titers (118). Although these results seem to support the use of plasmapheresis for AAV administration in seropositive individuals or for vector re-administration, the procedure is burdensome and results in transient suppression of the immunoglobulin in circulation. For this reason, two specific approaches were recently developed (119, 120). Both of them were based on the immobilization of an AAV capsid (AAV8 and AAV9 respectively) on a chromatography resin then used to specifically sequester anti-AAV antibodies from serum. These proof-of-concept studies clearly demonstrated the advantages of antigen-specific IgG depletion over plasmapheresis. However, the clinical development of this technology may prove complex and the demonstration of efficacy in large animal models is still missing.

An alternative to the use of plasmapheresis could be the reduction of circulating IgG with a bacterial protein, the IgG-degrading enzyme of Streptococcus pyogenes (IdeS) (121, 122). This cysteine endopeptidase is part of the bacterial arsenal to defend themselves from IgG-mediated opsonization (121). IdeS was originally proposed to prevent kidney rejection in HLA-sensitized individuals (123–125), and is now approved for use in this patients’ population (Idefirix^®^, Hansa Biopharma). The molecular mechanism of action is based on the very fast and efficient cleavage of human IgG into F(ab’)2 fragments and Fc (126, 127). Although the F(ab’)2 fragments still retains some neutralizing activity, in the absence of the Fc portion, they are rapidly eliminated from the circulation. One of the biggest limitations for the identification of the potential of IdeS in gene therapy with AAV is the lack of preclinical models to demonstrate its efficacy. IdeS is highly specific for human IgG, it does not cut murine IgG and it is only partially efficient towards monkey IgG. *In vitro* digestion of monkey IgG with IdeS resulted in the degradation of the vast majority of the IgG. However, single chain IgGs, intermediates of the digestion reaction with a neutralizing potential and not easily cleared from the circulation, were still present after overnight digestion (128). Recently, we demonstrated the efficacy of IdeS for the degradation of anti-AAV antibodies and the successful administration of AAV vectors in seropositive NHPs (128). In particular, IdeS pre-treatment improved liver transduction efficacy in seropositive NHPs both in the context of first administration in monkeys naturally exposed to AAVs and in a re-administration setting (128). Confirmation of the potential of IdeS in AAV gene therapy was further provided by the use of IdeZ, an IdeS homolog (111). Importantly, although the injection of the bacterial protein induced the formation of anti-IdeS antibodies, they were not neutralizing the protein function, and IdeS was able to degrade IgG in the presence of anti-IdeS antibodies both *in vivo* and *in vitro* (128). Another interesting property of IdeS is its capacity to cleave B-cell receptor and temporarily inhibit memory B-cell activation (129) thus providing a further advantage when it comes to the administration of vectors in non-naïve individuals. In conclusion, IdeS has the potential to enable AAV vectors administration in seropositive patients even in the context of repeated administration of the same vector. One limitation toward the clinical use of IdeS in this setting could be that the repeated administration of IdeS may trigger a hypersensitivity reaction. Ongoing work is trying to address this potential issue by developing new IdeS molecules with reduced immunogenicity to unlock the potential of this approach in autoimmune diseases, chronic transplant rejection, oncology and gene therapy.

An orthogonal approach to IdeS, based on a distinct bacterial protein, is the use of protein M, an IgG binding protein isolated in human mycoplasma (130). Very positive preliminary data on the use of this protein to reduce the neutralizing titers against AAV were communicated (131) although no published data are available so far.

## Conclusions

The overcoming of humoral immune response to AAVs is key to unlock the full potential of gene transfer with this vector.

The exclusion of patients from clinical trials based on the presence of antibodies against the gene therapy vector raises also important ethical questions. Different methods were developed to evaluate the neutralization activity against AAV vectors in patients (**Table 1**) , however limitations still exist and validation of those methods across laboratories is required to allow comparison of the data and equal access to the treatment starting from early-stage clinical research.

**Table 1:** Methods currently used to evaluate antibody response to AAV vectors.

Method	Neutralizing factor detected	Unit	Sensibility	Standardization possible	Advantages	Limitations	Used in clinical trials
**ELISA**	Binding Abs	Concentration or titer (1:x) where x corresponds to the higher dilution giving greater OD than cut-off	+	+	AAV serotype-independent Detection of different Ab classes	Do not reflect neutralizing activity Saturation effect; need to test several sample dilutions Availability of specie-specific secondary antibodies	Yes
**Dot-blot**	Binding Abs	% of max intensity signal	–	–	AAV serotype-independent Detection of different Ab classes	Complex assay Few samples tested	No
***In vitro* NAb**	Neutralizing activity	Titer (1:x) where x corresponds to the first dilution at which at least 50% inhibition of the reporter gene expression is measured	+/- Depending on reporter gene, cell line and multiplicity of infection (MOI) used	+/-	Detects neutralizing activity *in vitro* Can be used with any AAV serotype Can be used with any species (do not require a secondary antibody)	Low transduction efficacy may affect the results Do not discriminate IgG classes or neutralizing factors other than Ig	Yes Routinely
***In vivo* NAb**	Neutralizing activity	% of inhibition of reporter gene expression compared to control mice	–	–	Detect neutralizing activity *in vivo* Can be used with any AAV serotype Can be used with any species (do not require a secondary antibody)	Low throughput High variability Do not detect low levels/low affinity neutralizing factors	No

**Figure 2:**
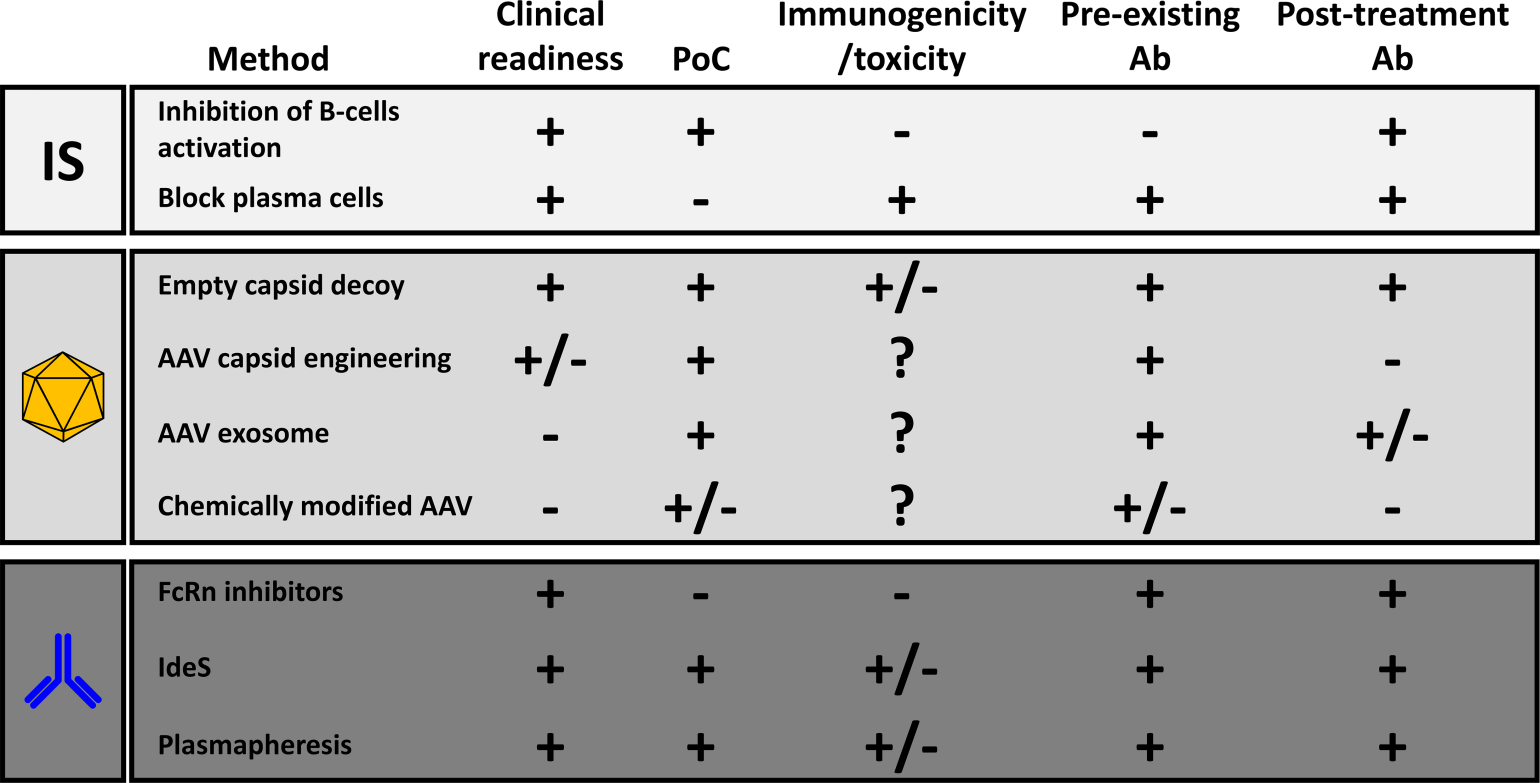
Comparative analysis of the different methods to reduce the anti-AAV humoral response. The last two columns refer respectively to the efficacy of the method in the reduction of the impact of pre-existing neutralizing antibodies or in the inhibition of the anti-AAV humoral immunity after treatment with recombinant vectors. PoC, proof-of-concept with AAV vector gene transfer; Ab, antibodies; FcRn, neonatal Fc-receptors; IdeS, Immunoglobulin G-degrading enzyme of S. Pyogenes.

Different methods were developed in the past to reduce the impact of anti-AAV NAbs (**Figure 2**). Among them, immune suppression strategies inspired to those developed in transplantation seem to be the one with the highest potential in the clinic. So far, clinical proof of their efficacy in the reduction of anti-AAV antibody titers has been obtained only in individuals that, being seronegative, were likely naïve for AAVs.

The modification of the surface of AAV through direct engineering of the capsid sequence, or vector shielding with empty particles, exosomes and through chemical modifications was also proposed to escape neutralization. These techniques can be used to prevent neutralization in seropositive individuals. However, after the first injection the generation of antibodies specific for those modified capsids is likely to prevent vector re-administration. AAV-exosome have the potential to achieve re-administration although data in relevant preclinical models are still missing.

A more specific alternative to immune suppression is to transiently reduce the levels of IgG, known to be the more important class of antibodies neutralizing AAV vectors, to create a window for the delivery of AAV vectors to target organs. The possibility to administer AAV vectors in seropositive monkeys through IdeS pre-treatment supports the hypothesis that by clearing circulating IgG a safe and efficient gene transfer can be achieved in the liver. Similar results were obtained with plasmapheresis in primates further confirming AAVs as vectors with a relatively low immunogenicity also in individuals with pre-existing immunity. Proof-of-concept of the efficacy of these approaches required adaptation due to the low activity of IdeS on monkey IgG and the small size of the animals. A clinical demonstration is likely required to demonstrate the full potential of these approaches.

Intravenous infusion is probably the route of administration where NAbs have the largest negative impact. Other routes of administration of AAV vectors, e.g. intravitreal or intrathecal are being tested in the clinic. For these routes, further studies are needed to better understand the impact of pre-existing NAbs on AAV gene transfer. The spatial constrains, the different composition and density of the fluids in these compartments and potential differences in the antibody composition of vitreous or cerebrospinal fluid may impact the efficacy of the different methods described in this review and they will possibly need to be adapted to the specificities of those compartments.

The administration of AAV vectors in non-naïve individuals is a potential concern when one of these strategies will reach the clinic. Immune responses to AAV vectors are unique in humans and it cannot be excluded that pre-exposed and naïve individuals may react differently to AAV vectors. This is particularly relevant for studies involving large doses of AAV vectors where toxicity was observed, possibly dependent on complement or platelets activation. Importantly, future studies of AAV administration in seropositive individuals will take advantage of the large clinical experience available on the control of AAV vector immunogenicity.

In conclusions, although all the approaches described in this review have the potential to reduce the impact of NAbs on tissue transduction, their efficacy was frequently demonstrated either with low neutralizing titers or through partial reduction of higher titers. Lower neutralizing titers are observed in individuals naturally infected by AAV and this is possibly the first population that will benefit from these approaches. In case of vector re-administration, the titers are in general more elevated and a combination of orthogonal techniques for the reduction of the impact of NAbs is potentially needed.

Despite the challenges imposed by the humoral immune response to AAV gene transfer, the knowledge on the immune response to AAV vectors and the technological advances make possible the clinical validation for some of these approaches. As we learned through the extensive clinical experience with AAV gene transfer, only a confirmation of safety and efficacy in humans could open the way to a generalized use of techniques to decrease the impact of humoral immune response on AAV gene transfer. The stakes are extremely high. On one hand, pre-existing immunity prevents seropositive patients to access a life-changing treatment. On the other hand, setting-up of protocols for safe and efficient AAV vector re-administration will completely change the paradigm of gene therapy, moving from a once-in-a-life treatment to a treatment on-demand. This is a fundamental step to ensure durability of the treatment and provide a better alternative to other treatment like small-molecules or protein replacement therapy.

## Author Contributions

D-AG, NT, CL, and GR wrote the manuscript. All authors contributed to the article and approved the submitted version.

## Funding

This work was supported by Genethon. GR was supported by a grant from the World anti-doping agency (WADA) and a grant from the IMI2-2019 Call of the Horizon 2020 framework program (ARDAT, ID 945473). D-AG was supported by Agence Nationale de la Recherche (ANR-15-CE15-0005-02), an ATIGE grant from Genopole (Evry, France) and Paris Île-de-France Region (DIM Thérapie Génique).

## Conflict of Interest

The authors declare the following competing interest: CL and GR are inventors in patents related to AAV gene therapy and the control of immune responses against AAV vectors.

The remaining authors declare that the research was conducted in the absence of any commercial or financial relationships that could be construed as a potential conflict of interest.

## Publisher’s Note

All claims expressed in this article are solely those of the authors and do not necessarily represent those of their affiliated organizations, or those of the publisher, the editors and the reviewers. Any product that may be evaluated in this article, or claim that may be made by its manufacturer, is not guaranteed or endorsed by the publisher.
